# The influence of the noradrenergic system on optimal control of neural plasticity

**DOI:** 10.3389/fnbeh.2013.00160

**Published:** 2013-11-13

**Authors:** Massimo Silvetti, Ruth Seurinck, Marlies E. van Bochove, Tom Verguts

**Affiliations:** ^1^Department of Experimental Psychology, Ghent UniversityGhent, Belgium; ^2^Ghent Institute for Functional and Metabolic Imaging (GIfMI), Ghent University HospitalGhent, Belgium; ^3^Department of Data Analysis, Ghent UniversityGhent, Belgium

**Keywords:** locus coeruleus, ACC, norepinephrine, plasticity, volatility, reinforcement learning, prediction error, learning rate

## Abstract

Decision making under uncertainty is challenging for any autonomous agent. The challenge increases when the environment’s stochastic properties change over time, i.e., when the environment is volatile. In order to efficiently adapt to volatile environments, agents must primarily rely on recent outcomes to quickly change their decision strategies; in other words, they need to increase their knowledge plasticity. On the contrary, in stable environments, knowledge stability must be preferred to preserve useful information against noise. Here we propose that in mammalian brain, the locus coeruleus (LC) is one of the nuclei involved in volatility estimation and in the subsequent control of neural plasticity. During a reinforcement learning task, LC activation, measured by means of pupil diameter, coded both for environmental volatility and learning rate. We hypothesize that LC could be responsible, through norepinephrinic modulation, for adaptations to optimize decision making in volatile environments. We also suggest a computational model on the interaction between the anterior cingulate cortex (ACC) and LC for volatility estimation.

## Introduction

Discovering the value of actions and environmental states is a key task for (animal or artificial) autonomous agents. When an environment changes its stochastic properties (e.g., the probability of obtaining food in a specific place), it is defined as volatile. In this case, the agent has to quickly adapt to new conditions, making its knowledge structures more plastic (e.g., increasing neural plasticity) and finding a new optimal decision strategy. A previous fMRI study proposed that the anterior cingulate cortex (ACC) is directly involved in volatility estimation and learning rate modulation (Behrens et al., 2007). However, more recent computational, electrophysiological and imaging studies (Alexander and Brown, 2011; Kennerley et al., 2011; Silvetti et al., 2011, 2013) suggest that ACC computes outcome expectations and the ensuing prediction errors (PEs) (i.e., the difference between outcome expectation and actual outcome). The estimation of volatility is a statistic of higher order than outcome expectation, as outcome expectation regards the probability of obtaining a specific outcome in the next future, while volatility regards the probability that the outcome-related statistics (e.g., reward rate) will change over time. In other words, outcome expectation (and related PE) deals with single events, while volatility estimation is about the environment’s statistical structure. Here we propose the locus coeruleus (LC) as an alternative candidate for volatility estimation and learning rate control (Jepma and Nieuwenhuis, 2011). This small brainstem structure that releases the neurotransmitter norepinephrine seems to be a promising candidate for a series of reasons. First, the LC is bidirectionally connected with a wide range of cortical and subcortical structures, providing a noradrenergic modulatory control. Norepinephrine can adaptively change neural parameters, such as the task-relevant selectivity (gain) of perceptual and premotor neural populations (Aston-Jones and Cohen, 2005a) or synaptic plasticity (LTP) (Katsuki et al., 1997; Izumi and Zorumski, 1999; Verguts and Notebaert, 2009). Second, earlier work suggested that pupil diameter is enhanced by large errors (single events) when subjects tried to predict numbers extracted from a specific probability distribution (state PE in absence of decision making), and correlates with subsequent updating of learning rate (Nassar et al., 2012). Pupil diameter is strongly correlated with LC activity (Rajkowski et al., 1993; Aston-Jones and Cohen, 2005a), and LC has been shown to regulate pupil size both directly (via iris dilator muscle) (Yoshitomi et al., 1985) and indirectly (sphincter muscle relaxation via inhibition of the Edinger-Westphal nucleus) (Loewy et al., 1973; Breen et al., 1983). More generally, unexpected events triggers an orienting response, with pupil size being one of its components (Nieuwenhuis et al., 2011). Third, a computational study (Yu and Dayan, 2005) suggested that phasic norepinephrine bursts can be associated with detection of unexpected events in changing environments.

**Figure 1:**
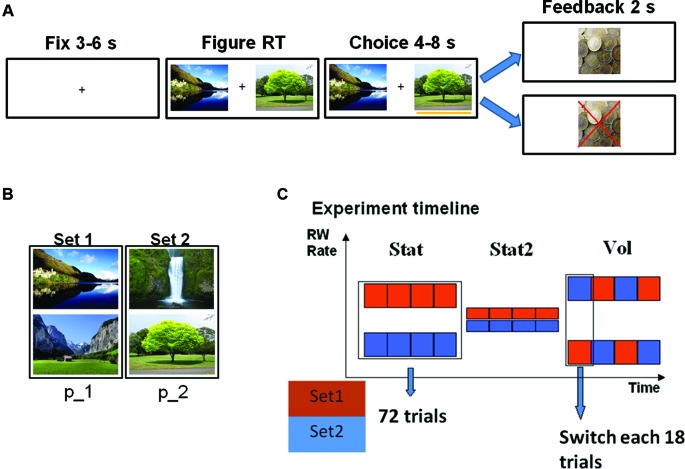
**(A)** Trial timeline (left rewarded, right unrewarded trial). Participants’ choice was communicated by appearance of a yellow bar under the selected figure. Time intervals indicate jittering. **(B)** Figure sets. Each set was assigned a specific reward probability (p_1 or p_2). **(C)** Experimental timeline showing reward rates for each figure set (color) as a function of time. Each square represents a block of 18 trials. During the volatile (Vol) environment, reward probabilities were switched between sets each 18 trials.

For all these anatomical and functional reasons, LC seems an excellent candidate for volatility estimation and optimizing performance in volatile environments, and can be studied by using pupil diameter. In order to investigate the role of LC in volatility estimation, we administered to a group of healthy participants a reinforcement learning task (Kennerley et al., 2011) (variant of 2-armed bandit task, Figures 1A, B) in three different statistical environments (SEs, Figure 1C): a stationary environment (Stat), a stationary environment with high amount of uncertainty (Stat2), and a volatile environment (Vol). During the task we continuously recorded pupil diameter. We thus investigated the relation between SE, LC activation, and learning rate. Finally, we propose a computational explanation on the origins of the LC response.

## Materials and methods

### Participants and behavioral task

We administered a probability tracking task (Kennerley et al., 2011) (Figure 1A) to 26 healthy volunteers, all students of Ghent University (five male). All the volunteers gave written informed consent to participate in the study. One participant was excluded for excessive blinking and another for evident problems in maintaining alertness during the task. During each trial, participants were asked to choose one of two pictures displayed on a screen. Each picture belonged to a set of two pictures; each set was linked to a specific probability of receiving a monetary reward (13 eurocents). Participants’ goal was to discover by trial and error which pictures were the most advantageous. Each SE consisted of 72 trials, for a total of 216 trials and duration of about 43 min. In the Stat environment the links between picture sets and reward probability did not change: one set led to a 70% reward rate (*p*_1_ = 0.7), while the other to 30% (*p*_2_ = 0.3). During Stat2 both choices provided a highly uncertain outcome (60% reward rate). In the Vol environment the reward rates were 90% and 10% and they switched between sets each 18 trials (so the 90% reward rate pictures became 10% reward rate pictures and vice versa). Before the actual task, subjects performed a training session of 72 trials in a Stat environment. The order of SE presentation was balanced between participants, as well as the link between reward probability and picture sets in Stat environment (in half of the participants Set 1 was the most rewarded). In order to match the reward rate of the Stat environment with the other two SEs, we introduced in this environment 10% of trials in which both pictures came from the low probability set. Consistent with previous studies (Behrens et al., 2007; Silvetti et al., 2013), in both behavioral and pupil size analysis, we excluded the first 18 trials at the beginning of each SE, in order to rule out spurious volatility due to the switch of contingencies between two environments. It is worth stressing that the excluded trials were those at the boundary between two successive SEs, and not between the probability switches inside the volatile SE.

### Learning rate estimation

Learning rate was estimated by fitting choices of individual participants with a Rescorla Wagner-Softmax selector system (Behrens et al., 2007). For each participant, the learning rate of the reinforcement learning agent was estimated by Maximum Likelihood Estimation (MLE). The temperature parameter was fixed at 0.015.

### Pupil diameter analysis

Pupil diameter was continuously recorded during the task by an Eye Link 1000 Tower Mount (SR Research) eye tracker, with a sampling rate of 1 KHz. Pupil time series were preprocessed to remove blinks, and then passed to a 2 Hz low-pass filter (MATLAB, The MathWorks Inc.). Each preprocessed time series was then segmented by EEGLAB software (Delorme and Makeig, 2004) using the onsets of participants’ choices and feedbacks, within a time window of 4 s after the onset. Each segment was baseline corrected (baseline: average signal from −500 to 0 ms to onset). Finally the average signal for choice period and feedback period was computed for each participant in order to perform a group analysis. Statistical analyses were performed using SPSS software. In order to investigate in which specific time bins of the choice period there was a significant difference between Vol and Stat2 condition, we ran a separate analysis on the pupil time course in this epoch. First, a paired *t*-test was calculated at each time point to determine the statistically significant (*p* ≤ 0.05) time bins. Given the large number of consecutive tests and the high amount of autocorrelation in the data, this can create false positives. Based on the autocorrelation and the number of time bins observed in the data, we used a simulation procedure with 5000 iterations to calculate the minimal length of a statistically significant interval (Guthrie and Buchwald, 1991). Only intervals where the number of consecutive significant time bins had at least the required length were considered to reflect a statistically significant difference in pupil width between Vol and Stat2.

Although no difference in reward rate was found between SEs, there was an increasing trend resembling the pupil grand averages. For this reason, we performed a further repeated measures ANOVA, after removing the variance explained by reward rate from the pupil diameter signal. A separate linear regression for both the choice and feedback period was calculated using each participant’s reward rate as the only predictor for pupil diameter. This yielded a pupil signal predicted by reward rate; this prediction was subtracted from the original pupil signal, thus resulting in the corrected (residual) signal.

### Pupil size-learning rate correlation

In order to test the hypothesis that norepinephrine levels (measured by pupil size) regulate learning rate at the behavioral level, we measured the between subjects correlation between pupil size and estimated learning rate. To remove between-subject differences in the overall level of these measures, we used the *Stat* SE as a baseline to create within-subject measures that represent the increase of learning rate and pupil size during the *Vol* SE. More precisely we computed

(1) $$\text{Corr}\left(Pupil_{Vol_{i}}-Pupil_{Stat_{i}},LR_{Vol_{i}}-LR_{Stat_{i}}\right)$$

where Corr(x,y) indicates Pearson’s linear correlation, and Pupil and LR indicate respectively pupil size and learning rate values for subject *i* during the SE *Vol* or *Stat*.

## Results

**Figure 2:**
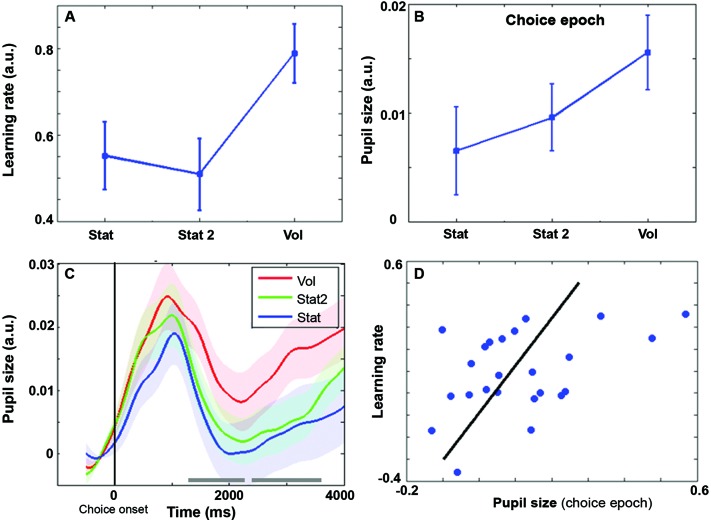
**(A)** Behavioral learning rate (± s.e.m.) as a function of SE. **(B)** Average pupil size during the choice epoch (± s.e.m.) as a function of SE. **(C)** Pupil size time course grand averages (baseline corrected) for choice epoch (± s.e.m.). Vertical bar: choice onset. Timeline in milliseconds. Grey horizontal bars indicate the time windows in which the difference between Vol and Stat2 is significant (cluster level family-wise corrected *p* < .05) **(D)** Scatter plot representing the covariation between pupil size and learning rate during the Vol period. Each data point is a single subject average. Regression line is shown in black.

Average reward rates were 59, 59, and 63% respectively for Stat, Stat2, and Vol, (*F*(2,46) = 3.11, *p* = 0.054). At the behavioral level, the learning rate (Figure 2A) was influenced by environmental volatility (*F*(2,46) = 4.69, *p* = 0.014). Learning rate was highest during the Vol environment (contrast Vol-Stat: *t*(23) = 2.49, *p* = 0.02), while no difference was found between Stat2 and Stat (contrast Stat2-Stat: *t*(23) = −0.43, *p* = 0.67).

Pupil diameter was computed in two time epochs of 4 s width, one time-locked to the choice and the other to the feedback onset. Repeated measures ANOVA revealed an interaction environment x epoch (*F*(2,46) = 7.96, *p* = 0.001). Remarkably, during the choice epoch, pupil diameter was wider in the Vol environment than in Stat2 and Stat (Figure 2B; main effect SE: *F*(2,46) = 5.73, *p* = 0.006; contrast Vol-Stat2: *t*(23) = 2.64, *p* = 0.015). Increased pupil size before actual feedback may be useful to prepare for increased learning in the upcoming feedback phase (see Discussion). Figure 2C shows the pupil size time course during the choice epoch. As shown, there are two large time windows in which the pupil diameter is significantly larger for Vol than for Stat2 condition. No difference between Stat and Stat2 was found during the choice epoch (Stat2-Stat: *t*(23) = 1.00, *p* = 0.33).

During the feedback epoch, we apparently found a reversed situation, with pupil diameter smaller in the Vol environment than in the Stat environment (main effect SE: *F*(2,46) = 3.21, *p* = 0.049; *t*-test Stat-Vol: *t*(23) = 2.54, *p* = 0.018), no difference was found between Vol and Stat2 (Stat2-Vol: *t*(23) = 1.70, *p* = 0.103). Like in the choice epoch, no difference between Stat and Stat2 was found (Stat2-Stat: *t*(23) = 0.74, *p* = 0.47). The difference between Vol and Stat condition during the feedback epoch most likely results from a baseline artifact. Indeed, pupil diameter signal was baseline corrected and pupil diameter just preceding the feedback onset was much wider for the Vol condition (last 500 ms of the plot in Figure 2C), creating a stronger baseline correction.

Although no significant difference in reward rates was found between SEs, the average reward rate in Vol environment was slightly higher than in the other two (63% vs. 59%). For this reason, we removed the variance explained by reward rate from the pupil diameter signal and recalculated the repeated measures ANOVAs for testing differences between SEs within each epoch (choice and feedback). After data correction for reward rates, we ran the same data analysis flow used for choice and feedback uncorrected data: results remained basically unchanged. The main effect of SE during the choice epoch was: *F*(2,46) = 3.67, *p* = 0.033; contrast (one-sided) Vol-Stat2: *t*(23) = 1.92, *p* = 0.034; contrast Vol-Stat: *t*(23) = 2.80, *p* = 0.01). During the feedback epoch, the main effect of SE was: *F*(2,46) = 3.75, *p* = 0.031; contrast Stat-Vol: *t*(23) = 2.77, *p* = 0.011, contrast (one-sided) Stat2-Vol: *t*(23) = 1.88, *p* = 0.036. Like for the uncorrected data, in both choice and feedback epochs, no difference between Stat and Stat2 was found (choice epoch: *t*(23) = 0.95, *p* = 0.35; feedback epoch: *t*(23) = 0.74, *p* = 0.47). Finally, Figure 2D shows a positive correlation between pupil size during the choice epoch and learning rate (*r* = 0.43, *p* = 0.034).

## Discussion

**Figure 3:**
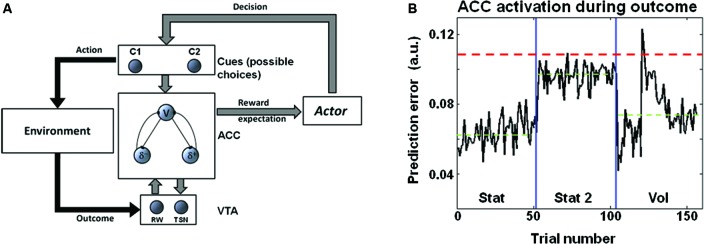
**(A)** Schema representing the RVPM. The system consists in a module simulating the ACC (that estimates reward expectations, V unit, and computes PEs, δ units), a module simulating the dopaminergic brainstem nuclei (VTA), a module making decisions on the basis of the ACC expectations (Actor module), and a module representing stimuli or possible actions (Cues module). Once the choice is made, the environment provides an outcome that is encoded by the VTA module, which delivers to the ACC module a dopaminergic reward signal. The VTA module receives recurrent connections from the ACC module, allowing dopamine shifting from reward period to cue period. **(B)** Results of RVPM simulations supporting the model, from Silvetti et al. (2013). The plot shows the ACC module PE signal (sum of all the units’ activity) as a function of trial number, in three different SEs. Although the average PE signal (green dashed line) is highest during Stat2 (highly uncertain environment), Vol environment triggers very strong phasic PE activity. This property can be exploited for volatility detection, e.g., the red dashed line indicates a possible threshold for volatility detection based on PE magnitude.

Pupil diameter during choice suggested that LC codes for environmental volatility. The norepinephrinic output increases before the outcome and thus, before the computation of the PE. In this way, this mechanism can modulate neural and synaptic functions, such as LTP, controlling neural plasticity for fast changing environments just before the actual PE is provided, and the synaptic connections are updated. Consistently, at the behavioral level, we observed an increased learning rate in the volatile condition (Figures 2A, D). It is worth noting that in an earlier study we have shown a different activation pattern in the ACC, namely stronger ACC activation in stationary highly uncertain environments (Stat2) (where the average PE is maximal) than in volatile environments (Silvetti et al., 2013). We propose that the LC processes cortical signals afferent from the mid frontal cortex (in particular from the ACC) (Jodo et al., 1998; Aston-Jones and Cohen, 2005b; Samuels and Szabadi, 2008) for the purpose of volatility estimation. In other words, LC could extract information about volatility level from the ACC signal by means of processing the time course and amplitude of PE signals. More specifically, a possible mechanism could consist in time integration of bursts of PE signals from ACC. Indeed, although in uncertain environments the average PE is higher than in volatile environments (Silvetti et al., 2013), in the latter very strong phasic PE activity occurs, due to the changing of statistical contingencies and the consequent strong violation of the ACC expectations. This theory is illustrated in Figure 3B. This figure shows the PE time course from the ACC simulation ran in Silvetti et al. (2013). In that study we administered to a neuro-computational model of ACC-VTA (the Reward Value Prediction Model (RVPM; Figure 3A)) a reinforcement learning task very similar to the one we used in this study (Figure 2 in the original study of Silvetti et al. (2013)). The RVPM executed the task in the same SEs we used here (Vol, Stat2 and Stat). Although the average PE was highest in the Stat2 condition (consistent with fMRI results from the same study), the volatile environment evoked the biggest bursts of ACC activation. These bursts could be a useful marker for volatility detection, a specific feature of PE signal that can be used by the LC in order to provide an explicit estimation of volatility. In summary, we hypothesize that surprise (ACC PE) and norepinephrine-dependent learning rate are combined together to generate a signal updating reward expectations. An alternative theory about learning rate control was proposed in the Predicted Outcome-Response (PRO) model (Alexander and Brown, 2011). In the latter work, the authors proposed that learning rate of action-outcome expectations is dynamically set directly within the ACC. This proposal is computationally elegant and parsimonious, nonetheless it misses to capture the findings about norepinephrinic activity and behavioral/synaptic plasticity (including the ones from the present work). By contrast, dynamic learning rate adjustment by LC not only allows to explain a wider set of experimental data, but it also implies the computational advantage of setting simultaneously the learning rate over several cortical/subcortical structures, due to the extent of norepinephrinic efferents.

Although link between pupil size, unexpected environmental states (single errors during a number magnitude task), and behavioral learning rate was previously suggested (Nassar et al., 2012), our study advances for the first time an explicit theory on the relationships between Reinforcement Learning, decision making, environmental volatility, LC activity and learning rate, inscribing it in a specific neuro-computational framework on cortical-subcortical structures related to Reinforcement Learning. Our theory is consistent with Yu and Dayan (2005) proposal that the environment can be assumed to be changed (i.e., volatile) if the level of norepinephrine crosses a (variable) threshold (determined by acetylcholine). In our conceptualization, ACC activation needs to exceed a threshold, but the underlying principle is the same. Assume a context has been changed, and update your learning speed, but only when the amount of PE is sufficiently high. Interestingly, our work provides also insight on the classical issue about the tradeoff between stability and flexibility (Carpenter and Grossberg, 1988). Indeed, volatility estimation and consequent adaptation by LC may allow preserving useful knowledge from local chance fluctuations of the environment (stability), but simultaneously allows updating knowledge when the environment actually changes (plasticity). A very recent work (O’reilly et al., 2013), integrating fMRI and pupillometry in a saccadic planning task, provided results that in part seem to challenge the findings of our and previous studies. Indeed, while the authors confirmed the role of ACC in PE computation for learning environmental contingencies, they also reported that pupil diameter was maximal after surprises that were not relevant for predicting future outcomes, and therefore they were not used by subjects to learn environmental contingencies. In computational terms, the pupil diameter was maximal in those trials (called one-off trials) where the learning rate was zero. The latter result deserves a short analysis. In the study of (O’reilly et al., 2013), the one-off trials were a minority of the general trial set (25%), were irrelevant for the formulation of expectations (they grossly violated the statistical contingencies of the environment), and were clearly distinguishable from other trials due to different perceptual features (color label). These aspects probably allowed the formulation of a top-down internal model that easily identified one-off trials and prevented them to influence behavior, treating them as surprising (infrequent) distracters. This would explain why they still evoked a robust LC response (odd-ball effect) without affecting behavior. By contrast, our paradigm investigated bottom-up (model free) contingency learning, where no symbolic cues were available to deduce the weight that a single trial should have in expectancies updating, and subjects had to formulate their expectations exclusively by evaluating the time course of the outcomes.

Our work relates to a broad literature indicating brainstem catecholaminergic nuclei as a system deputized to set neuronal parameters for optimizing behavior as a function of environmental contexts (Doya, 2002). Although the core of this perspective on brainstem nuclei is several decades old (Livingston, 1967), only recently the cooperation between neurobiological, behavioral and computational methods permitted partial disclosure of the neuro-computational meaning of this modulation. In conclusion, our findings corroborate the hypothesis that LC estimates environmental volatility, and it controls the learning rate to promote adaptation to fast changing environments by means of norepinephrinic neuromodulation. One future prospect will consist in upgrading the ACC-VTA neurocomputational model RVPM with the inclusion of an LC model and the simulation of ACC-LC-VTA interaction, in order to generate quantitative predictions that can be tested with fMRI or EEG.

## Author contributions

Massimo Silvetti: theoretical hypothesis, study design, data analysis, data collection, manuscript writing; Ruth Seurinck: study design, data analysis; Marlies E. van Bochove: study design, data collection; Tom Verguts: theoretical hypothesis, study design, manuscript writing.

## Conflict of interest statement

The authors declare that the research was conducted in the absence of any commercial or financial relationships that could be construed as a potential conflict of interest.
